# I Can’t Afford it Right Now, So it Doesn’t Matter” Structural Drivers of Viral Suppression Among Men Who Have Sex With Men: A Longitudinal Qualitative Approach

**DOI:** 10.21203/rs.3.rs-4001004/v1

**Published:** 2024-03-29

**Authors:** Emily Dove-Medows, Justin Knox, Mariah Valentine-Graves, Patrick Sullivan

**Affiliations:** University of Michigan–Ann Arbor; Columbia University; Emory University; Emory University

**Keywords:** structural racism, health inequity, viral suppression, men who have sex with men, care cascade, HIV

## Abstract

**Background:**

Racial disparities in outcomes across the HIV care continuum, including in viral suppression, have been observed among sexual minority men (SMM) living with HIV. Structural factors are drivers of these disparities, yet data is lacking at the individual level on how day-to-day experiences of these structural factors contribute to losing viral suppression, and what happens to SMM after loss of viral suppression, including whether they achieve viral suppression again over time.

**Method:**

We conducted longitudinal semi-structured interviews with a subsample of men living with HIV drawn from a larger cohort study. Three Black and 2 White SMM participated in a series of three interviews after they lost viral suppression, and then again at 6- and 12-months follow-up. The focus of the interviews was on experiences with structural issues (e.g., housing, transportation, employment, insurance) and their impact on HIV care.

**Results:**

Content analysis showed that multiple structural issues disrupted HIV care, particularly insurance, housing stability, transportation, and employment. Black SMM described experiencing multiple compounding structural barriers, and they struggled to achieve viral suppression again.

**Conclusions:**

These data show how SMM living with HIV are impacted by structural barriers to HIV care over time. Black SMM experienced multiple, compounding barriers, and these negatively impacted HIV care outcomes over time. Efforts to address long-standing HIV care-related disparities need to address the mechanisms of structural racism.

## Introduction

Structural racism–systems, institutions and processes that interact to produce and sustain inequities for racial and ethinc groups–has led to stark racial disparities in the US HIV epidemic (1, 2). Black Americans have an increased burden of HIV infection compared to White Americans including sexual minority men (SMM), a group that accounts for 2 out of 3 people living with HIV (PLWH) in the United States (3). Although research has focused on exploring the individual and societal factors that cause racial disparities in HIV incidence, less has been written about racial inequities in outcomes across the HIV treatment cascade (4–9). These inequities ultimately result in decreased levels of viral suppression among Black compared to White SMM (10–12). For example, in a large-scale study that included 423,651 SMM, 34% of White men achieved viral suppression compared with 16% of Black men (11). Lack of viral suppression results in increased morbidity and mortality among PLWH, and allows for potential onward HIV transmission (6, 13). Improving levels of viral suppression among Black SMM is necessary to address long-standing racial inequities in HIV burden (14).

While resarch has documented lower levels of viral suppression among Black SMM compared to White SMM, less is known about individual expereinces of the structual conditions (e.g. transportation, insurance, employment) that contribute to losing viral suppression, and what happens to SMM after loss of viral suppression and whether they achieve viral suppression again over time (10, 11). A large study of 651,811 PLWH found that Black men with at least one viral load test had lower rates of viral suppression compared to White men (41% and 56% respectively), and longer periods of time with higher viral loads (190 days in 12 months, 52%, and 149 days in 12 months, 41% respectively) (15). Other work has shown that multiple, overlapping structural factors contribute to these inequities, including employment, housing, insurance status, transportation, incarceration, racism and stigma (4, 16, 17).

Therefore, in the current study, using a longitudinal case study approach, we focused explicitly on how individual experiences of structural barriers and/or facilitatiors of viral suppression overlap and intersect with individual behaviors among SMM living with HIV in Atlanta, GA. Using qualitative data from a prospective cohort of Black and White non-Hispanic SMM living with HIV, we identified those who were either not virally suppressed at baseline or had a detectable viral load where previously suppressed and conducted in-depth interviews with those participants at that point, then again 6- and 12-months later.

## Methods

### Design and Sample

Data for this project were from EngageMENt, a mixed methods, prospective, observational, cohort study of 400 men who have sex with men (MSM) which was designed to describe HIV suppression and continuity of care among Black and White men living in Georgia from April, 2017 to June, 2018 (12). Inclusion criteria for EngageMENt were: assigned male sex at birth, 16 years or older, self-reported Black or White race, positive HIV status confirmed by HIV antibody screening, able to complete study activities in English, and with at least one male sex partner in the last 12 months. Enrolled men were followed for 24 months with surveys, qualitative interviews, and clinical visits. Survey and laboratory data were monitored to identify sentinel events, including lapses in care, antiretroviral adherence problems, detectable viral load in a previously suppressed participant, or lack of initial viral suppression. A subsample of 21 men experienced at least one sentinel event that was identified, and were invited to complete up to three in-depth interviews that focused on structural factors (housing, employment, transportation), and HIV care. From that group, 5 men were not virally suppressed at baseline or had a detectable viral load after being previously virally suppressed; these men were included in the current study.

### Data Collection and Analysis

Participants that experienced a sentinel event were invited to complete up to three in-depth interviews that focused on structural drivers of health including housing, status disclosure, and HIV care. Informed consent was administered in person at the study clinic, before the first enrollment visit. Interviews were conducted at baseline, 6, and 12 months after study enrollment and were conducted in-person by members of the study team. The interviews were conducted in a private setting and lasted 45 minutes to an hour each. Interviews were conducted using a timeline approach where participants and addressed clinical interactions and events (missed doses of antiretrovirals, HIV care), social events (anal sex, HIV serostatus disclosure with sex partners) and structural factors (access to care, transportation, housing, and employment). Participants selected for interviews received $60 cash at the completion of each interview.

Guided by the Bronfenbrenner’s socio-ecological model, EngagMENt explored several explanatory factors for inequities in HIV care and prevention. The interview guide included questions about structural drivers of health including access to care and insurance coverage, employment, and housing. Follow up interviews included a questions about these constructs as well as how they may have changed since the baseline interview. This study was reviewed and approved by the Institutional Review Board of Emory University.

### Qualitative Analysis

In-depth interviews were recorded, transcribed and de-identified and content analysis was conducted (19). A codebook was developed which incorporated concepts from the interview guide. Codes were further developed while reading and re-reading transcripts and coded using MAXQDA (20) according to study concepts and those that developed in an iterative process across transcripts, For example, additional codes pertaining to access to care, employment, and housing were created to facilitate a more in-depth analysis of these constructs. Coding queries were reviewed for all data relevant to the study aims and consensus was reached on codes after a collaborative review. A categorization matrix was developed based on the main study concepts and case summaries were created that included up to three interviews for each case. Within and across-case analyses were conducted. A total of fourteen interviews (four participants completed three interviews and one participant completed two interviews) were included in the analysis. The present study reports this research using the Standards for Reporting Qualitative Research framework. (18)

## Results

Among the men who participated in the qualitative interviews, five were identified who were either not virally suppressed at baseline or had a detectable viral load where previously suppressed, which included two White men and three Black men. The age range was 30–40 years with a mean age of 35. Participant incomes ranged from $0-$4,999 to >$75,000 annually. One of the participants had a college degree or higher, and the remaining four participants had less than a college degree.

Each participant experienced structural domains that operated as either a barrier or facilitator of viral suppression: insurance and access to care, housing, and transportation and employment. First, we will present these domains thematically, and describe the ways in which they operate in relationship among each other. Second, we will present two case studies that demonstrate how these domains are experienced by one individual and the impact on their ability engage in HIV care, and ultimately maintain viral suppression.

### Insurance and Access to Care

Access to both insurance coverage and health care functioned as a barrier to gaining and sustaining viral suppression for all participants, and was among the structural domains that was the most dynamic across all three interview timepoints for all participants. All participants experienced a lapse in insurance at least one timepoint that prevented access to HIV care and either led to or contributed to sustained loss of viral suppression. Change in employment over time further limited access to insurance and health care. Some participants experienced 90 day waiting periods as new, probationary employees which meant that even after becoming employed, at times following long periods of unemployment, they remained excluded from HIV care. One participant described during interview 2 the difficulties he experienced while trying to get back into HIV care.

I just couldn’t afford the medication, so just stopped taking it. I had a period where I had extra…so I tried to…take them, one this week, one next week, try to keep a little bit in my system. But once I ran out I just…couldn’t afford the medications. (Participant 1, Black, 35)

Despite working two jobs, he had outstanding medical debt from previous periods of being uninsured and had not investigated getting back into care. “*I haven’t even checked. I can’t afford it right now so it doesn’t matter*.” Another Participant described having to choose between maintaining employment while also needing to attend HIV care.

The first 90 days of any employment, they’re looking at your attendance. Spot on, they want you to be there every day and on time. I’m trying to make sure I’m staying employed. Honestly… I hate to say it, but it supersedes my healthcare sometimes. Without employment, I’m back where I began. I don’t want to go back there. I really don’t want to go back to that area. (Participant 3, Black, 30)

Participants also described a complex processes of planning for upcoming lapses in insurance brought about by both employment instability and housing instability.

They wanted to make sure I was mentally capable of sticking to that regimen. In the situation I was in, I was starting that decline of housing and stuff like that. I had just lost my job. I went in. I already had the appointment set. I just wanted to make sure I got my last doctor’s visit in before the insurance dropped off….That’s when he prescribed the antiretroviral. He didn’t really want to do that because once my insurance dropped off I wasn’t sure I was going to be able to keep it up. So, it was prescribed but I didn’t fill it. (Participant 3, Black, 30)

### Housing Instability

Housing instability was a predominant force in the lives of four out of the five participants across all three timepoints. Participants experienced periods of homelessness and often needed to move around to and from different family members or friends several times throughout the 14-month duration of the study. Lack of stable housing was a barrier to viral suppression, based on the practicality of having a place to store and take medications regularly.

There are times where I’m rushing to get to a bus. I left my medication at home. I’m not able to go back home because the last bus doesn’t leave until this time. I’m not going to make that bus. I’m trying to find a friend’s house to stay at. I don’t have the medication on me. Two days turns into three days without the medication.

Problems associated with the lack of stable housing impacted the ability to gain or maintain viral suppression. Physically getting to medications stored in unstable living situations was identified by several participants across all timepoints.

I know my meds are at home. I have a home. I know where they are…I know to take my meds. vs. before it was, oh, I left them at John’s house and John is not home. And now I got to wait. (Participant 3, Black, 30)

Participants also described situation the emotional toll of needing to depend on others for a place to stay. One participant *(White, 38)* described his ability to stay with others depended on his willingness to have sex. Others explained how the emotional work of being dependent on others for housing and how that impacted their relationships.

Depending on others for housing also created complications with getting to work. One participant described this frustration of dependance while being locked out of his temporary housing where his work badge and clothing were stored:

I sat at his door the entire day, knocking on the door, like please let me in. I got to get my work clothes. I got to get ready for work. I got to get my work clothes and my badge. And he wouldn’t let me in. (Participant 3, Black, 30)

Some participants reported during second or third interviews that were able to secure stable housing. This happened primarily thorough programs that were linked to their HIV care. Securing stable housing meant that participants were able to get to and from work on time, as well as store and regularly take their medication.

### Transportation and Employment

Lack of access to reliable transportation generated added a layer of complexity for participants trying to stay employed. With strict attendance policies and initial periods of probationary employment, participants described physically not being able to make it work from temporary housing and being at risk for losing work because of this lack of access.

But they changed [my hours] from 2:00 to 11:00. I can’t make it back in time enough to catch the bus to get to my brother’s house. So I would end up having to find places to stay after work at midnight. And it became to an issue where I would be late for work the next day and different things like that (Particpant 1, Black, 35)

Participants also described feelings of demoralization derived not having the freedom of movement and having to rely on public transportation:

I have a doctor’s appointment today at 3:40…I missed the one last Monday because of trying to get downtown with MARTA… I had to leave around 10:00 to get here in a sufficient amount of time or even to get to work in a sufficient amount of time. But they changed the bus schedules. I had to catch another bus. It was 12 minutes behind. So, it’s that type of situation. (Participant 3, Black, 30, interview 1)

All participants experienced a lapse in employment at one or more timepoints during the study which often set off a cascade of events leading to lapses in HIV care and periods of housing instability.

I have a doctor’s appointment today at 3:40…I missed the one last Monday because of trying to get downtown with MARTA… I had to leave around 10:00 to get here in a sufficient amount of time or even to get to work in a sufficient amount of time. But they changed the bus schedules. I had to catch another bus. It was 12 minutes behind. So, it’s that type of situation. (Participant 3, Black, 30, interview 1)

### Case Presentations

#### Individual who lost suppression and quickly regained viral suppression

Participant 2 was a 40-year-old White man. He temporarily lost suppression when he changed jobs, causing a short gap in insurance coverage. As a freelancer, he did not have full health insurance but had regular access to HIV care through the AIDS Healthcare Foundation. That coverage lapsed when he did not renew, yet he was able to remain suppressed because a family member prescribed his medications during that lapse.

Expired. I (was) just lazy (and) procrastinated. I was used to having really good health, so I didn’t really sweat it. As long as I was taking a medication regularly, I didn’t really sweat about it.

Participant 2 had access to his own vehicle during all interview timepoints and did not experience any housing instability. By interview 2, he had regained insurance through his job and was regularly accessed HIV care.

#### Individual who lost suppression and was not able to regain viral suppression

Participant 3 was a 30-year-old Black man. He recently started a new job but did not have insurance because there was a 60-day waiting period before he could enroll. His HIV medications cost $3000.00 without insurance but he was able to access HIV coverage through Ryan White insurance but this also came with challenges related to housing:

That’s when I stopped, I lost the meds. Basically I was on my last 30-day supply and dealing with all the moving back and forth, I was missing the appointments with my case manager, trying to reschedule.

During interview 1, he described the need to choose to work over accessing health care. Participant 3 did not have access to his own transportation which made getting to and from work and HIV care appointments difficult. During interview 1, participant 3 did not have his own housing after being evicted about three years ago. He was currently staying with different friends and relatives which often created difficulty maintaining viral suppression. He was in a four-year relationship that ultimately ended because of his HIV status. “*Once I found out that I was positive, we never had sex…So, we didn’t have sex for four years out of the five years…He was scared and didn’t know*.”

When participant 3 returned for the second interview, he lost his job due to ongoing attendance problems that were exacerbated by not having stable housing and transportation. Long and unreliable commutes often made him late or not be able to show up at all. There was an additional layer of stress of not knowing where you were going to stay each night and worry about how that may interfere with his ability to keep his job. He eventually did get stable housing but by that time, he had too many unexcused absences from work and lost his job the same day he finally secured housing.

I was like, hey, I am in between homes. I’m letting you know this right off the bat. There may be some days I come in a little bit late or behind, because I’m coming from this part of the city one morning and next night I’m coming from this part of the city. I’m trying to figure out the bus schedule, even if they have bus. I may have to walk a long period. Those different types of things. And some people don’t understand that way of living. They can just get up and go. But sometimes I have to get up or stay up late at night or be on my phone at work so that way I can have somewhere when I get off at midnight or 11:00.

## Discussion

This study explored in-depth and over time the experiences that SMM living with HIV had with structural barriers after they were identifies as not being virally suppressed or as having lost viral suppression. We found that several structural factors intersected to become either barriers which prevented the participants from regaining viral suppression or facilitators of achieving suppression and maintenance. The Black men in this study experienced several overlapping, multifactorial barriers that converged to prevent access to care both clinically (HIV care appointments) and at home (regular access to medications, serostatus disclosure). Furthermore, these barriers that led to loss of viral suppression, were also likely to persist over time.

Important research has shown how individual HIV-related risk behaviors are situated within social contexts (4, 5, 21). Our study builds upon this previous research and other work documenting racial disparities in HIV-related outcomes by helping to illuminate the individual, every day experiences of structural factors that drive inequities in HIV care and show how they persist over time (12, 22). The cases presented in this study show how several structural factors converge to negatively impact access to HIV care and treatment plans including antiretroviral therapy. One of the mechanisms through which structural racism works is an unequal distribution of access to opportunity (2). This is consistent with the overall rates of insurance for Black people in the United States who are more likely to not have access to health insurance compared to White people (23). Additionally, the participants experienced additional barriers to care including outstanding medical debt, so even after insurance is obtained, care remains out of reach (2). The ability to work steadily and achieve financial stability is an important facet of maintaining viral suppression (24, 25). Black men living with HIV in the United States are more vulnerable to unequal access to employment and experience more employment disruptions compared to White people (26). All participants in this study experienced a change in employment that interfered with HIV care at some time. This lack of financial stability and insurance coverage meant that their access to care was not only limited by structural and institutional factors but also compounded by inflexible working hours, living and working long distances from care.

Previous research has shown that SMM living with HIV are more likely to experience homelessness or housing instability (27, 28). The cases presented here show how housing instability compounded the complexity of access to care. Participants who lacked stable housing experienced difficulties getting to and from work and accessing their HIV medications while away from home. This study also illuminated an additional consequences of housing instability: status disclosure and condom negotiation. When the participants had their own private space, they expressed that they felt more open, willing, and safe do disclose their HIV status viral load, and to negotiate condom use. Without private space, many of the men discussed HIV status disclosure as something that happened covertly or as an unspoken understanding that left room for ambiguity.

Tenuous access to transportation hindered access to HIV care and employment. Being physically prevented from accessing places where HIV care is administered is part of a larger structural burden that disproportionately impacts Black men (29, 30). Racial residential segregation, a system where Black Americans are systematically excluded from certain neighborhoods, is based on racist historical and political practices and is one of the primary mechanisms of structural racism (31, 32). With this analysis, we provide first-person narratives of how a lack of transportation hinders access to health care, employment opportunities, and other services located in city centers, long distances from where participants live.

### Potential Interventions

The current study has implications for how to improve HIV care outcomes among SMM living with HV, particularly BSMM. Outreach to PLWH by community health workers has been identified as one successful strategy to improve HIV care outcomes and can be particularly meaningful for BSMM as they navigate intersectional stigma and the cycle of precarity that the individuals in this study described (33, 34). Improving relationships with clinical care providers and creating more welcoming clinical care settings is another important way to address health equity among BSMM. Indeed, structural competency, which emphasizes the need for deeper understanding of the structures—including racism—that inform clinical care encounters, has been identified as one way to advance health among BSMM (35, 36). When HIV providers have a more nuanced and culturally attuned understanding of the barriers to care outlined here as well as the underlying context of medical mistrust and inequity, they are better positioned to address the whole range of clinical care needs of BSMM that extend beyond just providing clinical care. Offering home based HIV care is another strategy that has the potential to HIV care inequities experienced among BSMM (37). For those study participants who struggled with either transportation or time barriers to physically getting clinics, home-based care is one way to increase access. Furthermore, combining home-based care with visits from trusted community health workers may improve both access and perceptions of individualized, culturally attuned care. Structural interventions, in particular those aimed at housing, could also help to explicitly address the factors that lead to racial inequities in HIV care outcomes and to achieve Ending the HIV Epidemic goals.

### Limitations

This study has certain limitations. Some participants were provided services (lab work, transportation) by the study which may have introduced some bias as they may have been more willing to participate given this benefit of the study. Also, there were only five participants in this study. It would have been stronger to include a larger, more diverse sample. However, fourteen interviews were conducted among these five participants, allowing us to understand in-depth how participants experienced structural barriers in their day-to-day lives, and how these were experienced over time. Further quantitative research is needed to evaluate the hypotheses generated by this in-depth qualitative research.

These limitations withstanding, this analysis shows how HIV care outcomes were shaped by structural barriers, and that these were inequitably experienced among the Black individuals who participated in this study. Structural barriers resulted in loss of viral suppression, and they often converged and persisted over time, particularly among the Black participants. The way that BSMM worked, travelled through the city, engaged in care, and in sexual encounters with other men was influenced by the lingering, pervasive histories of racism in the United States. This study shows how policy decisions, such as not to expand Medicaid or to provide inadequate housing support, work to disparately impact Black SMM and result in those who lose viral suppression to experience longer delays in being able to achieve viral suppression and maintain it over time (12).

## Conclusions

This study builds on prior quantitative work demonstrating racial disparities in viral suppression the role of structural barriers, and provides new insights about how the intersection of unstable housing, unemployment, access to care, operate in real time and conspire to form significant barriers in the HIV care continuum over time (12). While quantitative work has illuminated the relationships between these structural factors and inequities between Black and White people living with HIV(12), this longitudinal study demonstrates how individuals navigate these inequities day to day and over time. It also shows how precarious HIV care can be for Black SMM living with HIV in the US. Programs and policies aimed at improving HIV care outcomes also need to address racism both at individual and structural levels.
